# Italian Validation of the Scale of Psychological Abuse in Intimate Partner Violence (EAPA-P)

**DOI:** 10.3390/ijerph182312717

**Published:** 2021-12-02

**Authors:** Giulia Lausi, Benedetta Barchielli, Jessica Burrai, Anna Maria Giannini, Clarissa Cricenti

**Affiliations:** 1 Department of Psychology, Sapienza University of Rome, Via dei Marsi, 00185 Rome, Italy; jessica.burrai@uniroma1.it (J.B.); annamaria.giannini@uniroma1.it (A.M.G.); clarissa.cricenti@uniroma1.it (C.C.); 2 Department of Dynamic, Clinical Psychology and Health, Sapienza University of Rome, Via degli Apuli, 00185 Rome, Italy; benedetta.barchielli@uniroma1.it

**Keywords:** Italian population, psychological violence, self-report, violence against women, gender-based violence, domestic violence, assessment

## Abstract

Psychological and emotional forms of violence often represent a danger alarm and an important risk factor for other forms of intimate partner violence (IPV). Measuring psychological violence raises several issues of conceptualization and definition, which lead to the development of several assessment instruments; among them, the Scale of Psychological Abuse in Intimate Partner Violence (EAPA-P) showed good psychometric proprieties in a Spanish population and is used to identify which strategies are acted out to engage in psychological violence. The aim of the present study was to investigate the psychometric properties of the Italian version of EAPA-P among a group of Italian-speaking women (N = 343), thus evaluating its psychometric characteristics. Based on the English translation of the original Spanish version, an 11-item form of the EAPA-P was obtained, validity has been assessed through measures of emotion dysregulation, interpersonal guilt, conflict among partners and depression, anxiety, and stress symptomatology. Moreover, differences among groups were conducted to identify the capacity of the Italian version of EAPA-P to discriminate among women reporting experiencing psychological violence (N = 179), and who don’t (N = 150). Results showed an excellent internal validity, good correlations, and a good discriminatory ability of the scale. Strengths, limitations, and practical implications of the study have been discussed according to recent literature.

## 1. Introduction

Intimate partner violence (IPV) is a heterogeneous construct that includes several forms of violence—physical, sexual, economic, psychological/emotional, stalking—perpetrated by an intimate partner [1,2,3]. Although the reported prevalence of IPV varies considerably across studies [4,5,6,7,8,9], the findings are in agreement that the prevalence of the emotional/psychological form of violence is higher (5–91%) [5,6,7,10] than the physical/sexual form (3–59%) [5,6,7,11,12]. Psychological violence in IPV is frequently experienced in conjunction with other forms of violence, but can also occur alone and precede, or represent an important risk factor for physical/sexual violence [2,13,14,15,16].

Given the heterogeneity of IPV, it has been suggested to assess the occurrence of all forms of violence to detect the presence of emotional abuse or coercive control that may represent the first expression of maltreatment in intimate relationships and/or be indicative of a more severe form of IPV [3,17,18]. These considerations underlie the effort made in recent years in studying the consequences of different forms of IPV on women’s health. Particularly, several studies [17,19,20] highlighted experiencing psychological violence alone or in conjunction with other forms of violence leads to similar, if not more severe, mental health effects [3,13,21], even after exiting the abusive relationship [22,23]. Psychological violence, either alone or together with other forms of violence, would appear to be closely linked to emotional distress, presence of emotions such as shame and guilt [24,25,26] and difficulties in emotion regulation [27,28] or symptoms of stress, depression, and anxiety [3,19,22,29,30,31,32,33], suicidal ideation [19,34,35], and lower quality of life [36]. It is worth noting that while mental health effects in terms of psychopathology would seem to be common to all forms of violence, several studies [25,37], although not all of them [38], have found that other effects, such as guilt, would seem to be closely related to psychological abuse and would interact with it by amplifying the possibility of developing psychopathological symptoms such as post-traumatic ones.

### 1.1. Issues in Defining and Assessing Psychological Violence

Although in recent years there has been a “radical re-evaluation of the importance of emotional abuse in women’s mental health” [35,39], one of the reasons underlying the difficulty in comparing studies regarding the prevalence and the effects of psychological violence is based on issues of conceptualization, definition, and measurement of this form of violence [5,17,35,40]. This becomes evident from the use in the literature of broad terminology to refer to psychological violence, often also referred to as emotional abuse, coercion, psychological aggression [2,33,35].

A broad definition of psychological violence has been given by the European Institute for Gender Equality: *“Any act or behavior which causes psychological harm to the partner or former partner. Psychological violence can take the form of, among others, coercion, defamation, a verbal insult or harassment”* ([41], p. 45). Psychological violence is not considered as a unitary construct, but as a form of violence that contains within it several dimensions, such as dominance/isolation and verbal abuse [3,13,17,20,42,43], while other researchers proposed considering psychological violence on a continuum from forms of psychological aggression (e.g., insults) to more intense forms of coercive control [33,44,45]. On the other hand, Marshall [46,47] argued that psychological violence is more than just the dimensions that are identified from time to time, while the most important aspect is the use of the vulnerabilities or strengths of the victim as a weapon in the relationship. In general, the various definitions of psychological abuse include acts such as: yelling or screaming at a partner, insulting, humiliating even in front of other people, harassing, verbally threatening the victim, or loved ones with weapons or without, restricting the victim’s access to their social environment, financial resources, vehicle, or telephone, exploiting the victim’s vulnerability (e.g., undisclosed sexual orientation) [1,2,5,13,17,32,48,49]. Variations in the terminology and definitions of psychological violence have inevitably been reflected in the psychometric instruments developed to assess this form of violence [3,13,31,33]. Two main limitations of measurement tools in this field are the consideration of psychological violence as a discrete unit [13,17] and the stand-alone assessment of dimensions that fall under psychological violence [33,50,51]. Among the latter instruments are the Dominance Scale-DS [52], which provides a measure of dominance dimensions only. In contrast, most scales in this area are developed for the general measure of IPV or violence against women with a unique measure of the psychological form of violence, such as the Abusive Behavior Inventory [53], the Composite Abuse Scale [54,55], the Index of Psychological Abuse [43,56], the Partner Abuse Scale—Non-Physical [57], the Revised Conflict Tactics Scales (CTS-2) [51,58], the Safe Dates—Psychological Abuse Victimization [59,60], the Women’s Experiences with Battering [61,62,63]. For instance, a major limitation of CTS-2 [51,58], frequently used in studies of psychological violence, is the lack of adequacy and specificity in measuring the construct, not considering psychological violence as a multidimensional construct [31,64,65]. A minority of instruments, on the contrary, have also considered the different dimensions of the construct, such as: the Measure of Wife Abuse [50], which distinguishes between verbal and emotional violence; the Multidimensional Measure of Emotional Abuse [66,67] for measuring restrictive engulfment, hostile withdrawal, denigration, dominance/intimidation; the Profile of Psychological Abuse (MMEA) [68], which measures the dimensions of jealous control, ignore, ridicule traits, and criticize behavior; the Psychological Maltreatment of Women Inventory (PMWI) [42,69] that assesses the dimensions of dominance/isolation and Emotional/verbal; while other scales, such as the Subtle and Over Psychological Abuse Scale (SOPAS) [46], go beyond assessing distinct dimensions of psychological abuse by simply distinguishing between overt and subtle forms of violence.

### 1.2. The Scale of Psychological Abuse in Intimate Partner Violence (EAPA-P)

These are complemented by the Scale of Psychological Abuse in Intimate Partner Violence (EAPA-P) [70] which, based on the taxonomy developed by Rodriguez-Carballeira and colleagues [71] provides for the measurement of different strategies of psychological violence, from the most obvious to the most subtle, as outlined by Marshall [46]. EAPA-P begins, then, with the consideration that psychological violence, defined as *“continued application of strategies of pressure, control, manipulation and coercion with the purpose of dominating and subjugating the partner”* [70], can be enacted with direct strategies (influence on the emotions, cognitions, and behaviors of the partner) or with indirect strategies (control). This makes it possible to avoid a strict classification based solely on the tactics used by the perpetrator, thus allowing for a broadening of the acts themselves that are evaluated, considering instead the mode of violence acted out.

The validation of the original version of the EAPA-P involved a sample of 101 women with a mean age of 51.29 (SD = 13.04; range = 24–82) living in Spain and recruited through different municipal services that attend to these victims. Of these, 76% stated that they had experienced physical violence, 65% sexual violence, and 100% psychological violence. The 47 source items were formulated based on the taxonomy of psychological abuse strategies by Rodriguez-Carballeira and colleagues [71]. Following total item-scale, subscale, and corrected item-total correlations and exploratory factor analyses, 19 items were selected. A series of confirmatory analyses were conducted on this one, which indicated that the best model was the two-factor model: direct emotional abuse strategies and indirect emotional abuse strategies. The scale showed excellent internal consistency (α = 0.92) and convergent validity with the Subtle and Overt Psychological Abuse Scale [46], the Hospital Anxiety and Depression Scale [72], and the Davidson Trauma Scale [73].

The primary aim of this study is to develop an Italian version of EAPA-P [70] and to assess its psychometric properties. Furthermore, the concurrent validity of the EAPA-P was examined to provide additional information on the validity of this instrument.

*Research Question 1*: Investigate the psychometric properties of the Italian version of EAPA-P among a group of Italian-speaker women who reported to experience psychological violence

*Research Question 2*: Investigate how strongly the Italian version of EAPA-P correlates with other factors related to psychological violence

*Research Question 3*: Investigate the capacity of the Italian version of EAPA-P to discriminate among women who reported to experience psychological violence and who don’t.

## 2. Materials and Methods

### 2.1. Participants

A convenience sample of 452 participants, was recruited among adult population from Centers Against Domestic Violence as well as by voluntary participation through online recruitment within websites for listening and support for victims of violence. Anonymity and the right to refuse participation were guaranteed. The questionnaire was spread through the Qualtrics platform (Qualtrics.XM https://www.qualtrics.com/ accessed on 1 June 2021), including the requirement to fill in all questions in order not to have missing data. Participants could refuse their consent to fill in the questionnaire and drop out at any time. Inclusion criteria were to be a woman or self-identify as a woman (since the original version of the EAPA-P was validated on a sample of women), to be an Italian native speaker, to be of legal age, to have given their consent to the research and to fill in the entire EAPA-P scale. Three participants decided not to give their consent to participate in the study, and 106 participants did not fill in the entire EAPA-P scale therefore, the final sample comprised 343 participants. Sample characteristics were as follows: mean age was 26.62 years (SD = 6.33; range = 18–65); the sexual orientation was distributed as follows: 1.5% self-identified as asexual, 80.5% self-identified as heterosexual, 11.7% self-identified as bisexual, 1.8% self-identified as homosexual, 2.9% self-identified as pansexual and 1.5% self-identified as queer/other sexual orientation. 78.7% of the final sample referred they had never experienced physical violence from a partner, 18.4% had experienced it, and 2.9% chose not to answer the question; 43.7% did not experience psychological abuse from a partner, 52.2% had experienced it, and 4.1% chose not to answer the question (Table 1).

This study was conducted in accordance with the ethical standards of the Helsinki Declaration and was approved by the Institutional Review Board of the Department of Psychology of “Sapienza” University of Rome prot. N. 0000866.

### 2.2. Materials

An ad hoc online questionnaire was designed to collect personal information (i.e., gender, age, sexual orientation, history of physical and/or psychological abuse), and was distributed through different channels, such as Centers Against Domestic Violence and websites for listening and support for victims of violence. The questionnaire included the EAPA-P scale and the measures described in the next sections.

#### 2.2.1. Italian Translation of EAPA-P

Two independent researchers with excellent knowledge of English and psychological vocabulary translated the questionnaire from the English version of EAPA-P into Italian and then agreed on a common version. In this phase, particular care and attention were paid to avoid the presence of colloquial expressions, slang, or unintelligible or ambiguous phrases.

In the second phase, the common version was translated into English (back-translation) by a bilingual person with an extensive knowledge of psychological vocabulary; after correcting some translation inaccuracies, the final Italian version was obtained.

#### 2.2.2. Difficulties in Emotion Regulation Strategies-20 (DERS-20)

The Difficulties in Emotion Regulation Scales-20 [74] provides an assessment of emotional dysregulation across the following subscales: Non Acceptance (i.e., lack of acceptance of the emotional response); Impulse (i.e., difficulty in impulse control when experiencing negative emotions); Goals (i.e., difficulty distracting oneself from the emotion and performing alternative behaviors); Awareness (i.e., lack of emotional self-awareness); and Clarity (i.e., difficulty recognizing the emotion experienced); it is a self-reported questionnaire consisting in 20-items (e.g., “I pay attention to how I feel”), on a 5-point Likert scale, running from “Almost never” (1) to “Almost always” (5) (α = 0.921)

#### 2.2.3. Interpersonal Guilt Rating Scale (IGRS-15)

The Interpersonal Guilt Rating Scales-15s [75] is a measure used for assessing interpersonal guilt. The scale allows for the detection of three constructs: Survivor Guilt (i.e., a painful emotion felt when the person feels they have been more “fortunate” than others and views this as unfair), Self-Hatred (i.e., feeling of being inadequate, wrong, and not deserving of acceptance, love and happiness), and Omnipotence Guilt (which consists in two different sub-factors, the sense of separation/disloyalty and the omnipotent responsibility); interpersonal guilt is assessed through 15-items (e.g., “I feel uncomfortable feeling better off than other people”) on a 5-point Likert scale, running from “Not representative at all” (1) to “Completely representative” (5) (α = 0.874).

#### 2.2.4. Revised Conflict Tactic Scale (CTS-2)

The Revised Conflict Tactics Scale [51] is a 78-item measure of Intimate Partner Violence. The scale provides an assessment of both the victimization aspect (39 items) and the perpetration aspect (39 items) of violence. Consistent with the EAPA-P validation aim, only the scale that assesses the victimization aspects (i.e., negotiation, physical violence, psychological violence, physical injury, and sexual violence) have been used. Participants are asked to state how many times a specific situation happened in the previous year (e.g., “My partner threw something at me that could hurt”) on an 8-point frequency scale ranging from never to more than 20 times (α = 0.852).

#### 2.2.5. Depression Anxiety Stress Scales (DASS-21)

The Depression Anxiety Stress Scales [76] allows for the detection of three constructs: Depression, Anxiety, Stress. Depression includes dysphoria, hopelessness, devaluing life, lack of interest/involvement, anhedonia, and inertia; Anxiety relates to autonomic nervous system arousal, skeletal muscle effects, situational anxiety, and subjective experience of anxious affects; Stress relates to the presence of nonspecific arousal levels, difficulty relaxing, nervous excitement, irritability, agitation, hyperactivity, and impatience. Answers are assessed through 21 items (e.g., “I found it difficult to relax”) on a 4-point Likert scale ranging from “Did not apply to me at all” (0) to “Applied to me very much or most of the time” (3) (α = 0.954).

### 2.3. Data Analyses

Preliminarily, the CFA was conducted to verify the correspondence between the data obtained and the factorial structure proposed by the authors [70]. Once the values of the goodness of adaptation had been verified, an exploratory factor analysis (EFA) was carried out to identify the factorial structure in our sample. The analyses yielded a two-factor factorial structure. Based on this factorial structure, the items with a main saturation value lower than 0.4 and the presence of secondary saturations that were higher than half of the value of the main saturation [77] were removed. We obtained a 11-item structure on which an additional CFA and EFA were conducted to verify that the structure showed good fit indices and the presence of the original two-factor structure; afterward, reliability analyses were carried out.

To evaluate the concurrent validity of the EAPA-P with the other measures, the correlations between the EAPA-P, the DERS-20, the IGRS-15, the CTS-2, the DASS-21, and their subscales have been conducted. MPlus version 7 [78] was used to conduct the confirmatory factor analysis (CFA), while SPSS (statistical package version 25.0 (IBM, Armonk, NY, USA)) was used for the remaining analyses.

## 3. Results

### 3.1. Factorial Analysis

A maximum likelihood confirmatory factor analysis (CFA) was initially conducted to verify the correspondence between the data obtained and the factor structure proposed by the authors [70]. Given that the values of the indices obtained (χ2(151) = 785.854, *p* < 0.001; χ2/df = 5.20; RMSEA = 0.11; SRMR = 0.06; CFI = 0.86; AIC = 11265.57; TLI = 0.85) [79,80,81,82,83] weakly supported the original factor structure, an exploratory factor analysis was performed with maximum likelihood extraction method, and varimax with Kaiser normalization rotation method. Kaiser-Meyer-Olkin measure of sampling adequacy was used to examine the appropriateness of factor analysis: a value of 0.934 was obtained, indicting the appropriateness of the factor analysis. The Bartlett’s test of sphericity was significant (*p* < 0.001) meaning that our data set was suitable for data reduction. Based on the EFA factor matrix a two-factor solution was obtained. Some items showed a secondary saturation that was higher than half of the value of the main saturation (i.e., items 3, 6, 8, 9, 10, 13, 16) while item 2 did not saturate in any factor in an acceptable way (>0.40) [77]; therefore, these items were removed to maintain the solution’s goodness and a new scale consisting in two subscales and 11 items was obtained (Table 2 and Table 3). To verify the model fit measures, a new CFA was conducted. The fit measures were: the TLI and CFI were over the threshold value of 0.90 (TLI = 0.92; CFI = 0.94), the SRMR was good (SRMR = 0.047) and the RMSEA was marginal (RMSEA = 0.097).

### 3.2. Internal Consistency

To assess the internal consistency of our scale, a Cronbach’s alpha test was performed. Cronbach’s alpha was calculated for the total EAPA-P score and for each of the subscales (i.e., “Direct Psychological Abuse (PA) Strategies” and “Indirect PA Strategies”). The results indicated a high internal consistency, with an α of 0.90. The two subscales yielded an α of 0.88 for Direct PA Strategies, and an α of 0.87 for Indirect PA Strategies.

### 3.3. Concurrent Validity

The correlations among the EAPA-P scales ranged from 0.600 to 0.953; to examine concurrent validity, correlations among the EAPA-P and the other instruments (CTS-2, IGRS-15, DERS-20, DASS-21) have been conducted and are presented in Table 4.

Results showed that the total EAPA-P scale positively correlates with all forms of partner abuse (as measured by the CTS-2), with subdimensions of guilt (e.g., Self-Hate, as measured by the IGRS-15), with emotional dysregulation (DERS-20), and with symptoms of depression, anxiety, and stress (DASS-21), while negatively correlates with negotiation abilities (Negotiation subscale of CTS-2). The EAPA-P Direct PA Strategies subscale follows the same structure as the total scale. Finally, the EAPA-P Indirect PA Strategies subscale follows the same structure as the previous ones but the Omnipotence Guilt and Survivor Guilt subscales of the IGRS-15, the Non-Acceptance, Goals and Clarity subscales of the DERS-20 do not show significant correlations.

### 3.4. Differences among Groups

A Student’s t-test was conducted to compare the group of women who reported experiencing psychological violence (N = 179), those reporting not experiencing it (N = 150), the third group consisting of women who preferred not to report their experience (N = 14) was not considered due to its size. Statistically significant differences emerged between the group who reported having experienced violence and the group who reported not having experienced violence both on the entire EAPA-P scale and on both subscales; in all cases the group who reported having experienced violence showed higher scores on the various scales (Table 5). Cohen’s d was used to interpret the effect size, which was large for the total scale and for the direct strategies subscale, while medium for the indirect strategies subscale [84].

## 4. Discussion

This study aimed to investigate whether the Italian translation of the EAPA-P scale [70] could be an appropriate instrument to evaluate the presence of psychological abuse strategies in intimate partner relationships among women. Results showed a two-factor solution, like the original Spanish version. Some items (2, 3, 6, 8, 9, 10, 13, 16) showed a secondary saturation greater than half of the main saturation and were therefore removed. Analysis of these items showed that the content was too generic (e.g., item 2, “My partner insisted that in our relationship we should be above the pain and discomfort that each of us could feel”) or too extreme (e.g., item 13, “My partner controlled everything I did”) and at the same time, a different translation would not have corresponded to the original version. Despite the elimination of these items based on statistical criteria, the content analysis of the factorial structure suggested a retention of the original scale meaning. Exploratory and confirmatory analyses conducted to verify the new 11-item structure showed excellent internal validity and acceptable fit measures.

The correlations of the scale and respective subscales with the psychological constructs selected to assess convergent (CTS-2 except for the Negotiation subscale, IGRS-15, DERS-20, DASS-21) and divergent (Negotiation subscale of CTS-2) validity are in line with the authors’ initial hypotheses. Relationships between indirect strategies of psychological violence (measured by EAPA-P), omnipotent and survivor guilt (measured by IGRS-15), non-acceptance of one’s emotions, clarity of feeling, and ability to act goal-directed behaviors when upset (measured by DERS-20) did not show significant correlations. Although these constructs are not directly linked among them, we hypothesize that these are all factors most related to more explicit forms of violence.

Comparison between the group of women who reported experiencing psychological violence and the group of women who reported not experiencing it, showed significant differences in both the total scale and the two subscales, with higher values of psychological violence in the first group. This suggests that the EAPA-P, in addition to its acceptable psychometric properties, can correctly discriminate the presence of psychological abuse in the intimate partner relationship.

With respect to the validation of a scale developed in other countries, in this case Spain, there may be a cultural effect on IPV and psychological violence. Overall, studies within the European context have shown that one of the main cultural factors affecting victimization is the gender equality index (GEI). Specifically, women living in low/medium GEI countries are less likely to experience IPV than in high GEI countries. This counterintuitive association is known as the “Nordic paradox” [85], as Northern European countries are those with higher GEIs. This result can be explained by the fact that women living in countries with higher GEI are more able to recognize their exposure to IPV, thus increasing the possibility of detecting episodes of violence. Italy and Spain are neighboring Western European nations and this may explain why in addition to showing similar prevalence of IPV, they also show similar cultures, languages, and social identities [9,85]. Specifically, a study that compared adolescents with respect to psychological violence in Italy and Spain showed that the only descriptive differences between the two countries concerned power imbalance and social support, where the former emerged as higher and the latter lower in Italy, compared to Spain. However, no significant moderation related to the reference country was found, supporting a generalization of the results for the two samples [86].

Despite the importance of developing validated instruments for violence assessment, this study has some limitations. From a conceptual perspective, methodological progress toward defining and operationalizing psychological abuse is needed [35] in order to differentiate between frequencies and severity of the items: as suggested by Dokkedahl [33], items of psychological aggression are often together with items assessing controlling behaviors. The Italian version was obtained from the English version of EAPA-P, which was not the original scale language as done in previous research [87] following the guidelines for translation [88] to reduce the possible translation issues.

Moreover, it should be noted that double translation brings with it difficulties and risks in preserving the original meaning, although double-blind back-translation procedures reduce the possibility of errors, but do not void it. Finally, these assessment instruments are often developed and validated among female populations, despite there are no gender differences in the prevalence of psychological violence [33,89,90,91,92,93]. However, it is important to note that no agreement with respect to this trend appears in the literature. In fact, several studies have found a female [94,95,96,97] or male [98,99] prevalence in the perpetration of psychological violence. Given this, the possibility that there are differences with respect to the manner and meaning of violence acted out by the two genders should be considered [100]. For example, men would appear to experience more verbal forms of psychological violence, while women would appear to be more likely to experience controlling behaviors [89].

## 5. Conclusions

Despite all the limitations, however, this study provides a reliable and validated version of a useful instrument for understanding the assessment of psychological abuse in a female sample and could be broadening used in different research, such as investigate the relationship from psychological abuse and the possible effects on victims’ health. To the best of our knowledge, this is the first study in Italy aiming at the validation of an instrument of psychological violence.

The psychometric properties of the EAPA-P suggest that it can be used as an assessing instrument to evaluate the presence of controlling behaviors acted by a partner in female IPV-victims population. However, future studies should broaden the research and the validation also to male and transgender population, moreover, tools such as EAPA-P could be used to investigate the prevalence of psychological abuse phenomenon in different relationship patterns. In fact, psychological violence strategies may differ depending on whether the abusive partner self-identifies as a man or a woman; likewise the frequency and severity of abuse events may change according to the gender of the victim [101,102,103]. Similarly, future studies could assess the mental health consequences for victims of psychological partner violence, depending on whether it is perpetrated against men, against women, and within different relationship patterns [104,105].

EAPA-P has several clinical implications: firstly, it helps identify forms of psychological violence before they can escalate into other forms of violence; if health professionals can identify violence at its earliest stages, the long-term effects on a woman’s health can be minimized [17]. Secondly, the EAPA-P allows us to understand the different psychological violence strategies engaged in by the partner: understanding the mechanisms of violence used can be helpful for structuring increasingly effective support interventions. Psychological violence and its consequences on the population are increasingly getting attention both in research and in crime prevention and reduction of secondary victimization; having validated assessment instruments that can provide data on these phenomena can help authorities and institutions in creating support programs designed for specific communities. Finally, although Italy and Spain would seem to be similar countries and with the same risk factors influencing the possibility of suffering of psychological violence, further studies are needed to understand what differentiates, at a socio-demographic level, women who experienced psychological aggression and women who didn’t, and subsequently to compare the two countries.

## Figures and Tables

**Table 1:** Descriptive Statistics.

	N	M	SD
Age	343	26.62	6.33
		**Valid Percent**	
Sexual Orientation	Asexual	1.5 (N = 5)	
	Heterosexual	80.7 (N = 276)	
	Bisexual	11.7 (N = 40)	
	Homosexual	1.8 (N = 6)	
	Pansexual	2.9 (N = 10)	
	Queer	1.5 (N = 5)	
	Total	100	
Experienced Physical Violence?	No	78.7 (N = 270)	
	Yes	18.4 (N = 63)	
	Did not report	2.9 (N = 10)	
	Total	100	
Experienced Psychological Violence?	No	43.7 (N = 150)	
	Yes	52.2 (N = 179)	
	Did not report	4.1 (N = 14)	
	Total	100	

Note. M = Mean; SD = Standard Deviation.

**Table 2:** Exploratory Factor Analysis of EAPA-P (N = 343).

Item	Factor 1 (Direct Strategies)	Factor 2 (Indirect Strategies)
EAPA-P4	**0.813**	0.263
EAPA-P5	**0.755**	0.372
EAPA-P12	**0.742**	0.334
EAPA-P7	**0.730**	0.350
EAPA-P11	**0.685**	0.256
EAPA-P3	0.678	0.402
EAPA-P10	0.653	0.346
EAPA-P6	0.605	0.390
EAPA-P9	0.589	0.589
EAPA-P8	0.579	0.552
EAPA-P16	0.564	0.392
EAPA-P1	**0.534**	0.130
EAPA-P2	0.408	0.224
EAPA-P18	0.321	**0.811**
EAPA-P17	0.340	**0.807**
EAPA-P19	0.280	**0.751**
EAPA-P13	0.483	0.659
EAPA-P14	0.234	**0.568**
EAPA-P15	0.204	**0.560**
Average Variance Extracted	0.511	0.502
Composite Reliability	0.861	0.831
% of total variance explained		57.21

Note: Saturations that meet the inclusion criteria are highlighted in bold.

**Table 3:** EAPA-P scale, Italian and English version.

Factors	Item
Strategie Dirette di Violenza Psicologica **“Direct Strategies”**	Il mio/La mia partner ha dato un’interpretazione a modo suo degli eventi che ci hanno colpiti. **“My partner interpreted the things that affected us in his own way”**
	Il mio/La mia partner ha denigrato le mie iniziative o le mie proposte. **“My partner denigrated my initiatives or proposals.”**
	Il mio/La mia partner mi ha trattato/a con disprezzo. **“My partner treated me with scorn.”**
	Il mio/La mia partner mi ha mostrato affetto solo quando gli era utile. **“My partner was affectionate only when it was in his own interest”**
	Ho annoiato o infastidito il mio/la mia partner esprimendo i miei sentimenti. **“It bothered my partner when I expressed my feelings”**
	Il mio/La mia partner mi ha dato la colpa di cose per cui non avevo responsabilità. **“My partner blamed me for things I wasn’t responsible for”**
Strategie Indirette di Violenza Psicologica **“Indirect Strategies”**	Il mio/La mia partner ha controllato i nostri soldi e mi ha impedito di utilizzarli ogni volta che poteva. **“My partner controlled our money and restricted my use of it as much as possible”**
	Il mio/La mia partner mi ha impedito di lasciare liberamente la casa. **“My partner kept me from freely leaving the house”**
	Il mio/La mia partner mi ha impedito di stabilire legami con le persone a me vicine. **“My partner kept me from establishing relationships with the people around me”**
	Il mio/La mia partner mi ha impedito di svolgere attività che mi facevano stare bene o che mi piaceva fare. **“My partner kept me from doing activities I felt like doing”**
	Il mio/La mia partner ha provato ad allontanarmi dalla mia famiglia. **“My partner tried to keep me away from my family members”**

**Table 4:** Concurrent validity among EAPA-P Total Scale, EAPA-P Direct Strategies, EAPA-P Indirect Strategies, and other instruments.

	EAPAP_Total	EAPAP_Direct	EAPAP_Indirect
CTS_Negotiation	−0.298 **	−0.321 **	−0.173 **
CTS_Psychological Violence	0.684 **	0.671 **	0.521 **
CTS_Physical Violence	0.533 **	0.488 **	0.471 **
CTS_Sexual Coercion	0.543 **	0.506 **	0.464 **
CTS_Injury	0.475 **	0.430 **	0.431 **
IGRS_Omnipotence Guilt	0.119 *	0.144 **	0.038
IGRS_Separation/Disloyalty	0.169 **	0.169 **	0.124 *
IGRS_Self-Hate	0.285 **	0.311 **	0.155 **
IGRS_Survivor Guilt	0.159 **	0.167 **	0.101
DERS_NonAcceptance	0.146 **	0.177 **	0.046
DERS_Goals	0.135 *	0.159 **	0.053
DERS_Impulse	0.216 **	0.221 **	0.145 **
DERS_Clarity	0.195 **	0.232 **	0.070
DERS_Awareness	0.231 **	0.213 **	0.201 **
DASS_Depression	0.261 **	0.283 **	0.147 **
DASS_Anxiety	0.290 **	0.292 **	0.207 **
DASS_Stress	0.255 **	0.278 **	0.140 **

Note: ** Correlation is significant at the 0.01 level (2-tailed). * Correlation is significant at the 0.05 level (2-tailed).

**Table 5:** Comparison between groups on EAPA-P scale and subscales (*t*-test).

Independent Sample Test								
	*t*	df	Sig.	d	Multiple Comparisons	Mean Difference	M	SE
EAPA-P_Total	7.274	327	0.000 **	0.805	E vs. nE	4.75	16.49	6.35
EAPA-P_Direct	7.609	327	0.000 **	0.842	E vs. nE	3.58	10.69	4.60
EAPA-P_Indirect	4.525	327	0.000 **	0.501	E vs. nE	1.17	5.80	2.41

Note. ** *p* < 0.01. E = Experienced Psychological Violence; nE = non-Experienced Psychological Violence. M = Mean; SE = Standard Error.

## Data Availability

The data presented in this study are available on request from the corresponding author. The data are not publicly available due to privacy reason.
